# Heart rate response and recovery during exercise and dementia risk: a prospective UK biobank study

**DOI:** 10.1038/s41598-025-22299-2

**Published:** 2025-11-03

**Authors:** Yiran Wei, Yonglin Shan, Wenxiao Fan, Xin Huang, Jiahao Ding, Ming Mao, Qianying Liu, Minle Tian, Xuewei Li, Jie Lu, Hongli Chang, Yi Dong, Yifeng Du, Chengxuan Qiu, Xiaolei Han, Yongxiang Wang

**Affiliations:** 1 Department of Neurology, Key Laboratory of Endocrine Glucose & Lipids Metabolism and Brain Aging, Ministry of Education, Shandong Provincial Hospital affiliated to Shandong First Medical University, Jinan, Shandong P R China; 2 Shandong Institute of Brain Science and Brain-inspired Research, Medical Science and Technology Innovation Center, Shandong First Medical University & Shandong Academy of Medical Sciences, Jinan, Shandong P R China; 3 Hebei Province Key Laboratory of Integrated Traditional and Western Medicine in Neurological Rehabilitation, Hebei Province Cangzhou Hospital of Integrated Traditional and Western Medicine, Cangzhou, Hebei P R China; 4 Aging Research Center, Department of Neurobiology, Care Sciences and Society, Karolinska Institutet-Stockholm University, Stockholm, Sweden; 5 Department of Neurology, Shandong Provincial Hospital affiliated to Shandong First Medical University, No. 324 Jingwuweiqi Road, Jinan, Shandong 250021 P R China

**Keywords:** Cardiovascular health, Dementia, Cognition, Exercise stress test, Dementia, Cognitive ageing

## Abstract

The heart rate response/recovery (HRR) index, a common indicator for cardiovascular health during exercise, has been linked with neurocognitive disorders and mortality. However, the relationship between HRR index and dementia remains unknown. Electrocardiogram data from 46,348 middle-aged and older adults from the UK Biobank were analyzed following a standardized submaximal exercise stress test (15-second baseline, 6-minute exercise, and 1-minute recovery). The HRR index was calculated as the product of heart rate responses ratio during exercise (peak/resting heart rate) and recovery ratio (peak/recovery heart rate). We found that 519 participants were ascertained with dementia, including 232 with Alzheimer’s disease (AD) and 85 with vascular dementia (VaD) during a median follow-up period of 12.62 years. Higher HRR index and recovery ratio were significantly associated with a reduced risk of all-cause dementia (HR: 0.72, 95% CI: 0.54–0.94, *P* = 0.017; HR: 0.75, 95% CI: 0.57–0.99, *P* = 0.018), but not with incident AD or VaD, respectively. In addition, higher HRR index was significantly correlated with slower cognitive decline in processing speed, reasoning, and memory (β:2.39, 95% CI: 0.28–4.49, *P* = 0.027; β: 2.12, 95% CI: 0.26-4.00, *P* = 0.027; β: 0.03, 95% CI: 0.02–0.05, *P* < 0.001), while the recovery ratio was significantly correlated with slower cognitive decline in reasoning and memory (β: 1.91, 95% CI: 0.04–3.79, *P* = 0.045; β: 0.03, 95% CI: 0.01–0.04, *P* < 0.001). Higher HRR index and recovery ratios are associated with a decreased risk of incident dementia and appear to have beneficial effects on delaying cognitive decline. The possible mechanisms for this may involve autonomic function and neurovascular health.

**Supplementary Information:**

The online version contains supplementary material available at 10.1038/s41598-025-22299-2.

## Introduction

Dementia, a neurological disorder that affects over 47 million individuals globally, still lacks a cure. Expanding the window for intervention requires identifying novel targets before the emergence of key risk factors^1^. Evidence has accumulated that dementia, including Alzheimer’s disease (AD), shares common mechanisms with cardiovascular disease (CVD)^2,3^. Reduced cerebral blood flow^4,5^ and cardiac output^6,7^ are associated with cognitive dysfunction and dementia. Therefore, further research is required to better understand the vascular contributions to dementia pathology.

Vascular health has been evaluated by cardiac output^8^, aortic pulse wave velocity^9^, and endothelial function^9,10^. Heart rate (HR) recovery ratio after exercise testing has emerged as an indicator of impaired parasympathetic reactivation, which is associated with adverse cardiovascular outcomes^11,12^. HR response, influenced by chronotropic incompetence, is also an independent predictor of adverse CVD events and mortality^13^. The heart rate response/recovery (HRR) index that combines HR recovery ratio and HR response is a more simple and cost-effective measure to evaluate cardiovascular function. In recent years, HRR index has emerged as a predictor of CVD events and mortality^14^. HRR index has also been a better indicator of neurocognitive disorders^15^, which suggests that HR response and recovery post-exercise may be an important indicator of cognitive health. A previous from the Wisconsin Registry for Alzheimer’s Prevention (WRAP) prospective study with cognitively healthy late-middle-aged adults found that a graded exercise test may predict cognitive performance^16^. However, the association between HR profiles during exercise and dementia risk remains unexplored.

In this large-scale longitudinal cohort study from the UK Biobank, we hypothesized that acute cardiovascular adaptation to exercise, assessed *via* HRR, might be related with future risk of dementia, AD, and vascular dementia (VaD). We sought to examine the associations of HRR index and its individual components with dementia and cognitive decline over a 12-year follow-up period.

## Methods

### Study population and data source

The UK Biobank is a large-scale population-based prospective cohort study that includes 502,493 individuals aged 40 to 69 years at the time of recruitment. Upon enrollment, participants provided electronically signed consent and were invited to undertake baseline assessments at one of 22 centers across 2006–2010. All analyses were performed in accordance with the relevant guidelines and regulations, including the Declaration of Helsinki. Data collection was extensive, involving touchscreen questionnaires, interviews, physical measurements, health records, and biological samples. Further details about the cohort and study protocol are available on the official UK Biobank webpage (http://www.ukbiobank.ac.uk/wp-content/uploads/2011/11/UK-Biobank-Protocol.pdf).

**Fig. 1:**
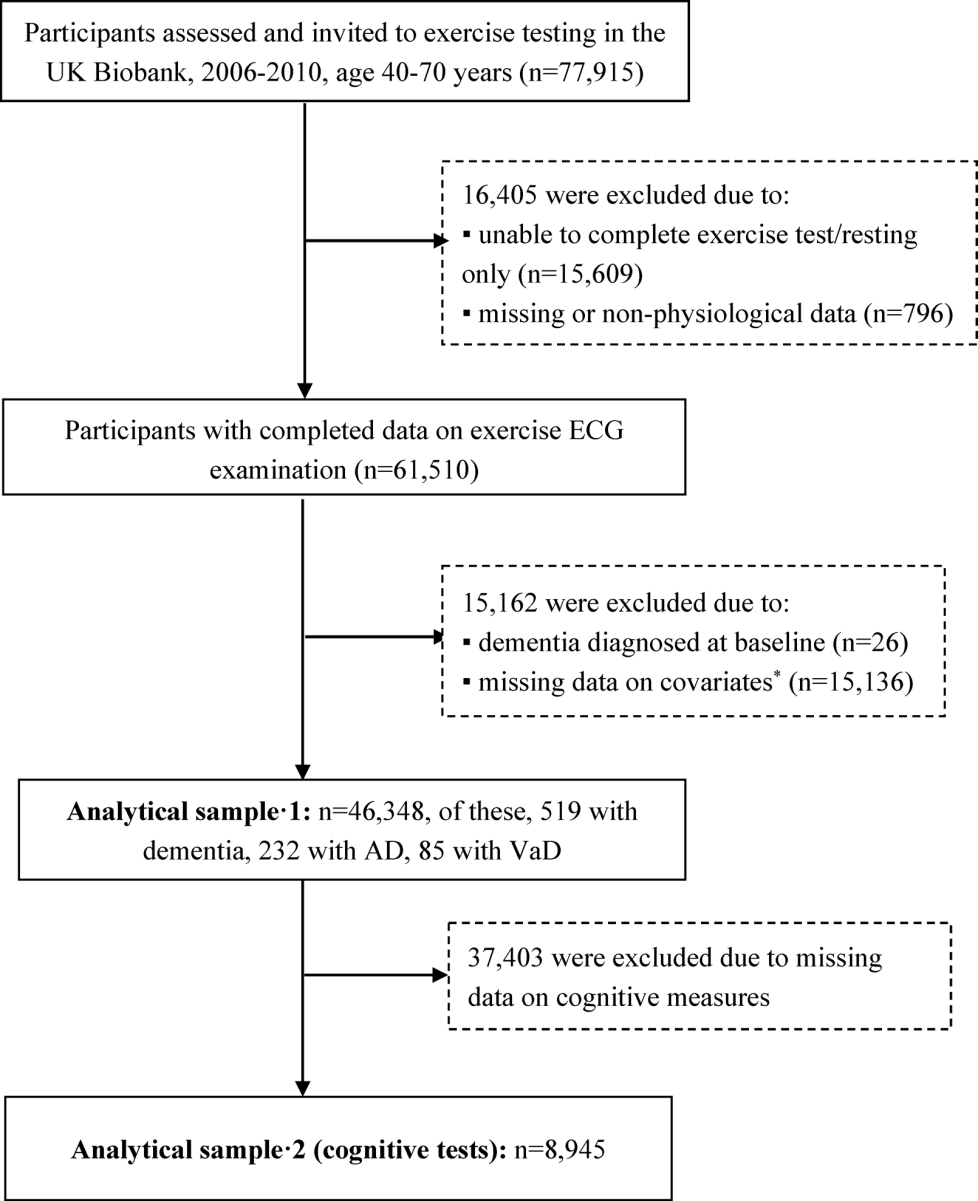
Flowchart of study participants.* AD* Alzheimer’s disease, * VaD* vascular dementia, * ECG* electrocardiogram.*The number of participants with missing values was 397 for ethnicity, 74 for Townsend deprivation index, 33 for body mass index, 189 for smoking, 32 for drinking, 11,471 for physical activity, and 2,940 for cardiovascular disease risk.

Between August 2009 and December 2010, 77,915 community-based participants from the United Kingdom were recruited to participate in an exercise stress test^17,18^. Of these, we excluded 16,405 participants who were unable to complete the exercise test or only had a resting ECG assessment. Participants with missing data on heart rate (HR) or with resting or recovery HRs below 40 beats per minute (bpm) or above 200 bpm (as these values were considered unlikely to be physiological), and those who were unable to achieve a HR higher than their resting HR (defined as a response ratio, calculated as peak HR achieved divided by resting HR, of less than 1) were also excluded (*n* = 796). We further excluded participants due to dementia at baseline or missing data on covariates (*n* = 15,136). Finally, 46,348 participants were included for the current analysis. For participants who remained dementia-free at the follow-up, we censored them until December 2022, the latest date available in the records. Additionally, we conducted a subgroup analysis of the correlation between the HRR index and cognitive tests. Participants in this subgroup had to complete baseline cognitive tests and at least one follow-up cognitive test. Eventually, 8,945 participants (19.30%) were included in this analysis. Figure 1 shows the flowchart of the study participants.

The UK Biobank was approved by the UK National Health Service Research Ethics Committee (references 16/NW/0274, 11/NW/0382, and 21/NW/0157). Written informed consent was obtained from all participants.

### Assessment of HRR index

Exercise tests were supervised by experienced physicians with the assistance of experienced nurses. Participants were assigned to carry out the individualized, submaximal bicycle protocols to increase the number of participants with exposure information and reduce the risk of adverse health events during exercise testing. The testing protocol and maximal desired participant effort were calculated from the risk category (determined from cardiovascular CVD risk factors), and the predicted maximal workload was determined from age, height, resting HR, and sex. Exercise testing was performed using an incremental ramp cycle ergometer test. The electrocardiogram (ECG) and HR were registered during the exercise test. HR was assessed by normal R-waves from ECG recordings using the modified Aristotle algorithm for QRS detection, consistent with previous studies^19,20^. The mean resting HR in this study was operationally defined as the pre-exercise value, while the peak HR during exercise represented the maximal achieved rate. Additionally, the recovery HR was determined at 1 min following cessation of exercise. In order to gauge a participant’s HR responses from rest to peak and subsequent recovery, regardless of preset workload, we determined: (1) response ratio = peak HR achieved/resting HR; (2) recovery ratio = peak HR /recovered HR (lowest HR in final rest phase); and (3) HRR index = response ratio × recovery ratio. Higher HRR index represented greater response and/or recovery. HRR index was categorized into low (≤ 25 percentile), average (25–75 percentile), and high (> 75 percentile) groups^15^.

### Diagnosis of dementia

Dementia was identified using the three-character International Classification of Diseases 10th revision (ICD-10) codes, which summarize the distinct diagnosis codes recorded for a participant across all their hospital inpatient records. This includes first incidents (Category 1712) and algorithmically defined outcomes (Category 47) in UK Biobank^21^. ICD-10 codes F00, F01, F02, F03, and G30 were used for all-cause dementia, ICD-10 codes F00 and G30 for AD and ICD-10 code F01 for VaD.

### Assessments of cognitive function

Cognitive tests were first administered *via* a touchscreen interface in the UK Biobank assessment centers at the baseline visit and repeated at the subsequent 3 follow-up visits. The detailed information is described on the website (https://biobank.ndph.ox.ac.uk/showcase/label.cgi?id=100026). All participants completed baseline assessments between 2006 and 2010. These tests offered a comprehensive evaluation of various cognitive domains, i.e., processing speed (reaction time), reasoning (fluid intelligence/reasoning), memory (prospective memory), and executive function (pairs matching)^22^. Prospective memory was assessed by giving participants an instruction that they had to remember later in the assessment, and scored as 1 if the participant remembered the instruction of their first try or 0 if not. For reaction time, fluid intelligence, and pairs matching tests, the raw cognitive test scores provided by the UK Biobank were standardized into z-scores for analysis, where higher values represent better cognitive ability.

### Covariates

Demographic information, including age (Field 21022), sex (Field 31), ethnicity (Field 21000), education (Field 6138), and Townsend deprivation index (Field 189), were obtained during the initial assessment. Educational attainment was self-reported and categorized as either college-level or non-college-level. Smoking status (Field 20116) was categorized as never, former, or current; alcohol consumption (Field 1558) was categorized as < 4 drinks per week or ≥ 4 drinks per week. Physical activity, quantified as the summed metabolic equivalent minutes (MET-min) per week for all activities (Field 22040), along with body mass index (BMI) (Field 21001), were integral components of the analysis.

The CVD risk score was calculated by counting the number of concurrent presence of hypertension, high cholesterol, diabetes, ischemic heart disease, and peripheral vascular disease, with the score ranging 0-5^15^. Additionally, we accounted for use of medications known to affect HR, such as beta-blockers and non-dihydropyridine calcium channel blockers (Field 20003) in our analysis.

### Statistical analysis

Descriptive analysis was conducted using one-way ANOVA for normally distributed continuous variables, Kruskal-Wallis test for non-normally distributed continuous variables, and the chi-square test for categorical variables (frequencies). Descriptive characteristics for the entire study group are presented as means with standard deviations (SD) for normally distributed quantitative variables and as medians with interquartile ranges for non-normally distributed variables, stratified by the three HRR groups. Categorical variables are reported as frequencies and percentages. The predictors included in this study were the HRR index and its individual components (resting HR, recovery HR, response ratio, and recovery ratio)^15^.

Cox regression models were used to estimate the longitudinal associations of HRR index and its individual components during exercise with risk of dementia. Model 1 was adjusted for age, sex, education, ethnicity, and deprivation; and Model 2 was additionally adjusted for physical activity, BMI, smoking, alcohol intake, CVD risk score, and use of HR-control medications. Cox proportional hazards regression analysis yielded estimated hazard ratios (95% CI). The Schoenfeld residuals test was used to confirm that the assumption of proportional hazards was satisfied. Linear mixed-effects models were applied to assess the associations between individual components of exercise HRR and cognitive changes in reaction time, fluid intelligence, and pairs matching. To improve the comparability of variables specifically for this analysis, we applied standardized z-score and subsequently multiplied all standardized values by 1000 to enhance readability and interpretation of the results. Additionally, generalized linear mixed models were performed to examine the association of HRR with prospective memory by the R package glmmTMB^23^. In sensitivity analyses, we used the Fine-Gray subdistribution hazard models to assess the potential impact of death on the observed associations between HRR index and incident dementia, while considering death during the follow-up period as a competing event.

We tested the statistical interactions of HRR index with age (< 65 vs. ≥65 years), sex (male vs. female), education (college/university degree or other professional qualification vs. non-college/university degree or other professional qualification), CVD risk score (none vs. at least one), physical activity (lower vs. higher), and use of any HR-control drugs (yes vs. no) on the risk of dementia by simultaneously entering the independent variables and their cross-product term into Model 2, in which HRR index was included as a continuous variable. Time-to-event was calculated as the time interval in years between the date of HRR assessment and the date of dementia onset.

All analyses were conducted using the R Statistical Software for Windows (version 4.4.1, R Foundation for Statistical Computing, Vienna, Austria). A two-tailed *P*-value < 0.05 was considered statistically significant.

## Results

### Characteristics of study participants

A total of 46,348 individuals from the UK Biobank were included in this study. Among those, the mean age was 55.95 years (SD, 8.1) and 50.8% were males. After a median follow-up of 12.62 years, 519 participants were identified with dementia, of which 232 cases were AD and 85 cases were VaD. The HRR index was categorized into low (≤ 1.89), average (1.89–2.58), and high (> 2.58) groups. Compared to participants with high or average HRR index, individuals with low HRR index were older, male, less likely to have attended college, a higher BMI, more frequent alcohol consumption, lower levels of weekly physical activity, and more likely to have elevated CVD risk and use of HR control medications (all *P* values < 0.01) (Table 1).

**Table 1:** Characteristics of study participants by the heart rate response/recovery index group (*n* = 46,348).

Characteristics	Total sample (*n* = 46,348)	HRR index group			*P* value
		Low (*n* = 11,587)	Average (*n* = 23,175)	High (*n* = 11,586)	
Age, years	56.38 (8.12)	58.66 (7.58)	56.43 (8.06)	54.00 (8.08)	< 0.01
Male, n (%)	22,912 (49.43)	6,886 (59.43)	11,076 (47.79)	4,950 (42.72)	< 0.01
College attendance, n (%)	18,201 (39.27)	4,133 (35.67)	9,136 (39.42)	4,932 (42.57)	< 0.01
Ethnic, white, n (%)	43,016 (92.81)	10,986 (94.81)	21,560 (93.03)	10,407 (90.37)	< 0.01
Townsend deprivation index	-1.27 (2.88)	-1.25 (2.89)	-1.33 (2.86)	-1.19 (2.90)	0.11
BMI, kg/m^2^	27.00 (4.40)	28.98 (4.70)	27.00 (4.22)	25.42 (3.71)	< 0.01
Alcohol consumption, n (%)	10,404 (22.45)	2,854 (24.63)	5,197 (22.43)	2,353 (20.31)	< 0.01
Smoking status, n (%)					< 0.01
Never	25,616 (55.27)	5,780 (49.88)	12,980 (56.01)	6,856 (59.17)	
Previous	16,667 (35.96)	4,506 (38.89)	8,230 (35.51)	3,391 (33.93)	
Current	4,605 (8.77)	1,301 (11.23)	1,965 (8.48)	799 (6.90)	
Physical activity level, MET-min^a^	2760.78 (2671.32)	2589.67 (2630.91)	2750.16 (2668.07)	2953.16 (2705.46)	< 0.01
CVD risk score^b^	0.49 (0.78)	0.74 (0.92)	0.45 (0.74)	0.31 (0.62)	< 0.01
HR-control drugs^c^, n (%)	1580 (3.41)	512 (4.42)	738 (3.18)	330 (2.85)	< 0.01
Resting HR, bpm	70.92 (11.46)	80.98 (10.98)	70.51 (8.62)	61.66 (8.31)	< 0.01
Peak exercise HR, bpm	112.98 (13.68)	110.31 (13.92)	112.68 (13.07)	116.25 (13.97)	< 0.01
Recovery HR, bpm^d^	81.85 (13.76)	90.23 (13.57)	82.05 (12.04)	73.09 (11.68)	< 0.01
Response ratio	1.62 (0.23)	1.37 (0.10)	1.60 (0.12)	1.90 (0.20)	< 0.01
Recovery ratio	1.40 (0.18)	1.23 (0.07)	1.38 (0.10)	1.61 (0.18)	< 0.01
Incident dementia, n (%)	519 (1.12)	197 (1.70)	245 (1.06)	77 (0.66)	< 0.01

Data are mean (standard deviation), unless otherwise specified.

Abbreviations: BMI, body mass index; bpm, beats per minute; CVD, cardiovascular disease; HR, heart rate; HRR, heart rate response/recovery; MET, metabolic equivalent.

^a^Physical activity level: Summed MET-minutes per week for all activities.

^b^The CVD risk score (range 0–5) was based on the presence of hypertension, high cholesterol, diabetes, ischemic heart disease, and peripheral vascular disease.

^c^HR controlling medications was beta-blockers and non-dihydropyridine calcium channel blockers.

^d^Recovery heart rate was defined as the heart rate measured 1 min after cessation of exercise.

### Associations between individual components of exercise HRR index and incident dementia, AD, and VaD

After controlling for age, sex, education, ethnicity, and deprivation, per 1-SD increment of HRR index was significantly associated with 10% lower risk of all-cause dementia (HR = 0.90, 95% CI: 0.82–0.99, *P* = 0.040). As a categorical variable, individuals with average (HR = 0.82, 95% CI: 0.67–0.98, *P* = 0.039) and high (hazard ratio = 0.73, 95% CI: 0.56–0.95, *P* = 0.035; *P* for linear trend = 0.02; Table 2) HRR indexes had significantly lower risk for all-cause dementia compared with low HRR index individuals. These associations remained statistically significant in model 2 when additionally controlling for lifestyle factors, cardiometabolic risk factors, and use of HR-control medications (Table 2). As the continuous measures, recovery ratios were significantly associated with lower risk of all-cause dementia (per 1-SD increment HR: 0.90, 95% CI: 0.81–0.99, *P* = 0.049). For categorical measures of recovery HR, the risk of dementia was 23% lower in high recovery ratios group (HR = 0.77, 95% CI: 0.56–0.99, *P* = 0.045) and 19% lower in average recovery ratios group (HR = 0.81, 95% CI: 0.67–0.98, *P* = 0.032) compared to low recovery ratios group. However, the HRR index and recovery ratio were not significantly associated with AD and VaD (Table 2).

**Table 2:** Associations of exercise heart rate response/recovery index with incident dementia (*n* = 46,348).

Exercise HRR group	Hazard ratio (95% confidence interval)					
	All-cause dementia (*n* = 519)		Alzheimer’s disease (*n* = 232)		Vascular dementia (*n* = 85)	
	Model 1	Model 2	Model 1	Model 2	Model 1	Model 2
HRR index						
Continuous ^a^	**0.90 (0.82**, 1.00)^*^	**0.90 (0.81**, 1.00)^*^	0.93 (0.80, 1.08)	0.91 (0.78, 1.06)	1.00 (0.79, 1.26)	1.03 (0.82, 1.30)
Categorical ^b^						
Low (1.05–1.89)	1.00 (reference)	1.00 (reference)	1.00 (reference)	1.00 (reference)	1.00 (reference)	1.00 (reference)
Average (1.89–2.58)	**0.82 (0.67**, 0.98)^*^	**0.83 (0.68**,** 0.99)** ^*^	0.93 (0.70, 1.23)	0.92 (0.69, 1.23)	0.64 (0.40, 1.03)	0.70 (0.43, 1.14)
High (2.58–9.73)	**0.73 (0.56**, 0.95)^*^	**0.72 (0.54**, 0.94)^*^	0.80 (0.53, 1.20)	0.76 (0.50, 1.16)	0.94 (0.52, 1.71)	1.02 (0.55, 1.90)
*P* for trend	**< 0.01**	**0.01**	0.29	0.72	0.48	0.21
Recovery ratio						
Continuous ^a^	**0.90 (0.81**, 0.99)^*^	**0.89 (0.80**, 0.99)^*^	0.99 (0.86, 1.14)	0.97 (0.84, 1.13)	1.04 (0.83, 1.31)	1.09 (0.86, 1.37)
Categorical ^b^						
Low (1.00-1.28)	1.00 (reference)	1.00 (reference)	1.00 (reference)	1.00 (reference)	1.00 (reference)	1.00 (reference)
Average (1.28–1.49)	**0.81 (0.67**, 0.98)^*^	**0.82 (0.67**, 0.99)^*^	1.06 (0.79, 1.42)	1.06 (0.78, 1.42)	0.59 (0.36, 0.94) ^*^	0.62 (0.38, 1.01)
High (1.49–3.24)	**0.77 (0.59**, 0.99)^*^	**0.75 (0.57**, 0.99)^*^	0.97 (0.65, 1.44)	0.93 (0.62, 1.40)	0.87 (0.48, 1.57)	0.93 (0.50, 1.73)
*P* for trend	**0.02**	**0.02**	0.97	0.80	0.30	0.47

Model 1 was adjusted for sociodemographic variables (age, sex, education, ethnicity, and deprivation). Model 2 was additionally adjusted for lifestyle factors (physical activity, body mass index, smoking, and alcohol intake), cardiovascular disease risk score (high blood pressure, cholesterol, diabetes, ischemic heart disease, and peripheral vascular disease), and use of heart rate control medications.

^a^ Per 1-SD increase.

^b^ HRR index was categorized into low (≤ 25 percentile), average (25–75 percentile), and high (> 75 percentile) groups.

Abbreviation: HRR, heart rate response/recovery.

^*^P < 0.05.

Significant values are in bold.

Similar to the results of the HRR index, recovery HR was significantly associated with increased all-cause dementia risk, whereas the association with AD or VaD was not statistically significant. Resting HR and response ratio showed no significant associations with dementia, AD, or VaD (Supplementary Table 3). There were no interactions of the HRR index and recovery ratio with sex, age, education, physical activity, or CVD risk score.

### Associations between individual components of exercise HRR index and cognitive decline

Controlling for age, sex, education, ethnicity, deprivation, physical activity, BMI, smoking, alcohol intake, CVD risk score, and use of HR-control medications, a lower HRR index at baseline was significantly associated with a faster decline in processing speed, as assessed by the reaction time tests (βs = 2.386, *P* = 0.026), memory, as assessed by the prospective memory (βs = 0.034, *P* < 0.001), and reasoning, as assessed by the fluid intelligence (βs = 2.124, *P* = 0.026). Moreover, a lower recovery ratio at baseline was associated with a greater rate of decline in reasoning (βs = 1.914, *P* = 0.045) and memory (βs = 0.027, *P* < 0.001) (Table 3).

**Table 3:** Associations between exercise heart rate response/recovery index and cognitive decline (*n* = 8,945).

Cognitive measures (outcome)	β coefficient (95% CI), average annual change in cognition	
	Model 1	Model 2
Reaction time ^a^		
HRR index × time	**2.375 (0.271**,** 4.480)** ^*^	**2.386 (0.281**,** 4.490)** ^*^
Recovery ratio × time	2.098 (-0.018, 4.214)	2.107 (-0.009, 4.223)
Fluid intelligence ^a^		
HRR index × time	**2.159 (0.293**,** 4.025)** ^*^	**2.124 (0.258**,** 3.990)** ^*^
Recovery ratio × time	**1.948 (0.071**,** 3.825)** ^*^	**1.914 (0.039**,** 3.790)** ^*^
Paris matching ^a^		
HRR index × time	-1.273 (-4.011, 1.464)	-1.248 (-3.986, 1.489)
Recovery ratio × time	-1.277 (-4.030, 1.475)	-1.224 (-3.977, 1.528)
Prospective memory		
HRR index × time	**0.034 (0.019**,** 0.049)** ^***^	**0.034 (0.020**,** 0.049)** ^***^
Recovery ratio × time	**0.027 (0.012**,** 0.041)** ^***^	**0.027 (0.012**,** 0.041)** ^***^

^a^Standardized z-score and subsequently multiplied all standardized values by 1000 were used to enhance readability and interpretation of the results.

Model 1 was adjusted for sociodemographic variables (age, sex, education, ethnicity, and deprivation); Model 2 was additionally adjusted for lifestyle factors (physical activity, body mass index, smoking, and alcohol intake), cardiovascular disease risk score (high blood pressure, cholesterol, diabetes, ischemic heart disease, and peripheral vascular disease), and use of HR-control medications.

Abbreviations: HRR, heart rate response/recovery; CI, confidence interval.

^*^P < 0.05, ^***^P < 0.001.

Significant values are in bold.

### Sensitivity analyses

We employed the Fine-Gray hazard models to examine the associations of baseline HRR index with incident dementia by considering death during the follow-up period as a competing event (Supplementary Table 1). The HRR and recovery ratios are marginally associated with decreased risks of all-cause dementia in Table 2. These results suggest that the competing risk of death may attenuate the direct association between the HRR index and dementia risk. Additionally, we have compared the baseline characteristics between participants in the current analytical sample (*n* = 46,348) and those who were excluded due to missing data on covariates (*n* = 15,136) (Supplementary Table 2). Our results showed that compared with the participants in the analytical sample, those who were excluded were less educated, had higher resting heart rate, peak heart rate, recovery heart rate, response ratio, recovery ratio (all *P* < 0.05), and marginally higher dementia incidence (1.31% vs. 1.12%), though HRR indices showed no significant difference.

## Discussion

In this large-scale longitudinal population-basted study, we revealed that a higher HRR index and recovery ratios during exercise were associated with a decreased risk of dementia in middle-aged and older adults. Moreover, higher HRR index and recovery ratios were linked to delayed cognitive decline in processing speed, reasoning, and memory.

Previous studies from UK Biobank study have shown that the HRR index was predictive of a neurocognitive disorder^15^ and an elevated resting HR was associated with an increased risk of dementia^24^. As a dynamic measure, the HRR index is easily calculated as the product of HR response and recovery ratios, offering a simple yet comprehensive assessment of overall cardiac regulation. While there is some evidence linking irregularity of heart beats and dementia^25,26^, there is a paucity of studies examining the prognostic value of HRR during exercise stress testing. To the best of our knowledge, this is the first large-scale prospective study examining the link between HRR during exercise and dementia in middle and older age over almost 12 years of follow-up. HR response to exercise and recovery from exercise provide complementary information about cardiac adaptability. Notably, a robust HR response during exercise does not necessarily ensure a rapid recovery of HR afterward^15^. The key novel finding from the current study is that dynamic changes in HRs from resting to peak exercise and recovery, rather than the average rates themselves, are most important in assessing future vulnerability to dementia. Notably, our study found that higher HRR index and recovery ratios were linked to a lower dementia risk, emphasizing the protective role of dynamic heart rate changes.

In addition, our study showed that an increased HRR index and recovery ratio were significantly correlated with cognitive decline in processing speed, reasoning, and memory, highlighting a key focus on domain-specific cognitive functions. The Cross-sectional study of aged adults with a wide range of CVD conditions (*n* = 47) has shown that reduced HR recovery was correlated with poorer cognitive performance, which were quite similar to our results^27^. By contrast, data from the study of cognitively normal, late-middle-aged participants found that individuals with lower HR responds had poorer cognitive performance, but not on HRR during exercise, which was inconsistent with our findings^16^. The inconsistent findings across studies might be partially attributable to cohort differences and the assessment of differences in the sample sizes as well as the younger age of participants in the current study compared to the previous one. Future studies are needed to clarify these discrepancies using larger, more diverse cohorts and standardized methods to assess HR dynamics and cognitive outcomes.

The mechanisms underlying the association between the HRR index during exercise and risk of dementia are not fully understood. A low HR response to exercise, characterized by a peak HR barely above the resting HR with a response ratio being close to 1, is identified as chronotropic incompetence. This condition is believed to indicate beta-adrenoreceptor dysfunction^13^. Conversely, post-exercise diminished HR recovery, where the lowest recovery HR remains near the peak HR resulting in a recovery ratio close to 1, is considered a marker of reduced parasympathetic reactivation and autonomic nervous system imbalance^28,29^. Autonomic dysfunction has been recognized as a key etiological factor, primarily due to negative effects on cardiac regulations. The failure of autonomic function is believed to be caused by cholinergic depletion which augments the inadequacy of cerebral perfusion on a long-term basis. Additionally, cerebral hypoperfusion contributes to white matter hyperintensities (WMHs), a key feature of AD pathology^30,31^. Tian et al.^32^ recently used Mendelian Randomization to show evidence Cardiac regulation for causal relationship between autonomic function and WMHs. Observational studies showed that heart rate variability (HRV), which reflects the balance between sympathetic and parasympathetic tone, is associated with WMHs. HRV may influence white matter structure through autonomic dysfunction, and these structural brain changes are associated with cognitive impairment. Additionally, previous studies demonstrate that physical fitness, hydration, and sleep patterns modulate autonomic regulation, critically influencing heart rate variability (HRV) and cardiovascular adaptability^33–35^. Future studies integrating real-time monitoring of these factors may clarify how transient autonomic fluctuations shape long-term dementia risk trajectories. Regarding the cognitive domains, evidence suggests that the association between HR reactivity and cognitive ability is specific for fluid reasoning and this association may be strongly driven by the influence from the sympathetic^36^. Additionally, findings from previous research indicate that the initiation of a reaction time task triggers a reflexive transition from vagal activation to vagal inhibition^36,37^.

Previous studies are similar to our findings. For example, some studies showed that coronary heart disease, heart failure, and atrial fibrillation were associated with an increased risk of all-cause dementia, while results for AD, VaD were inconsistent^38–40^. These inconsistencies could result from the specific pathological mechanisms of AD and VaD are independent of systemic cardiovascular effects^41,42^. Notably, a study reported that cerebral microbleeds are a common feature in both AD and VaD, creating a vicious cycle that accelerates cognitive decline^43^.

A longitudinal study including 7,522 community-dwelling participants with four years of follow-up found that the association between cardiometabolic multimorbidity and cognitive impairment was attenuated after adjusting for death as a competing risk, though the risk direction remained aligned with underlying pathological mechanisms. Since the HRR index reflects autonomic function, its significant association with heightened dementia and mortality risks makes them competing endpoints. Moreover, within limited follow - up periods, mortality events may cut short HRR’s long-term effects on dementia. Thus, extended longitudinal observation is needed to confirm potential associations. The excluded participants exhibited significantly higher resting HR and recovery HR compared to the analytical sample, alongside marginally elevated dementia incidence. These findings align with established studies: higher resting HR is associated with increased risk for dementia and faster cognitive decline independent of CVDs in elderly populations^2^, and higher recovery HR (indicating post-exercise diminished HR recovery) reflects autonomic nervous system imbalance. Notably, sensitivity analyses stratified by sex and education level revealed no significant subgroup differences, supporting the robustness of our primary findings. The exclusion of these individuals due to missing covariate data may have underestimated the true prevalence of dementia and, to some extent, the strength of associations between clinical, biological, neuroimaging factors and dementia.

Strengths of this study include the population-based design with a large cohort and a long follow-up period from midlife to late life. We could control for a broad range of health-related factors and health conditions as potential confounders, which were assessed from epidemiological, clinical, and biological aspects by local health care professionals. Nevertheless, the study has several limitations. Firstly, using hospitalization data as the primary source may miss undiagnosed or subclinical cases, potentially underestimating the association. Second, study participants were mostly whites, and findings may not be generalizable to populations from different socio-geographic and ethnic origins. Additionally, while useful in large - scale studies, these assessments may not capture the full complexity of cognitive function or subtle early impairments. We recommend supplementary cognitive measures to enhance future research. And the current analysis did not comprehensively account for potential confounding factors including genetic predisposition, systemic inflammatory markers, or neuroimaging evidence of cerebral small vessel disease. This incomplete adjustment for biological covariates may introduce residual confounding, thereby warranting cautious interpretation of the observed associations. More studies are warranted to validate these results and further understand the mechanisms linking HRR index with dementia and cognitive decline.

In conclusion, our study contributes to the growing body of evidence highlighting the significance of HR response and recovery during exercise as important markers of dementia risk. A high HRR index was associated with a reduced risk of all-cause dementia, while a lower HRR index at baseline predicted faster cognitive decline in processing speed, reasoning, and memory. These findings underscore the critical role of HRR in maintaining cognitive function and suggest that future research should explore if interventions to improve HRR, like exercise or medication, can change dementia risk.

## Supplementary Information

Below is the link to the electronic supplementary material.

Supplementary Material 1

## Data Availability

This work has been conducted using the UK Biobank Resource. This project corresponds to the UK Biobank application ID 91982. The UK Biobank is an open access resource and bona fide researchers can apply to use the UK Biobank dataset by registering and applying at http://ukbiobank.ac.uk/register-apply/. Further information is avail-able from the corresponding author upon request.
