# Supplementary material for: Heart rate response and recovery during exercise and dementia risk: a prospective UK biobank study
Source: Sci Rep. 2025 Nov 3;15:38362. doi: 10.1038/s41598-025-22299-2 (PMC12583445; doi:10.1038/s41598-025-22299-2)
Supplement: Supplementary file 1 — Supplementary Material 1 [file 41598_2025_22299_MOESM1_ESM.docx]

**Supplementary materials**

**Heart Rate Response and Recovery during Exercise, Cognitive Decline, and Incident Dementia: a Prospective Cohort Study of 46,348 UK Biobank Participants**

Wei Y, et al.

Contents

**Supplementary Table 1.** Associations of individual components of exercise heart rate recovery with incident dementia: Fine-Gray models.

**Supplementary Figure 1.** Risk analysis of individual components of exercise heart rate recovery quartiles for incident dementia over time

**Supplementary Table 2.** Characteristics of study participants by inclusion and exclusion status in the analytical sample.

**Supplementary Table 3.** Individual components of exercise heart rate recovery and their associations with incident dementia.

**Supplementary Table 4.** Subgroup analyses of dementia risk with recovery ratio and exercise heart rate response/recovery index.

**Table S1.** Associations of individual components of exercise heart rate response/recovery index with incident dementia: Fine-Gray models ^a^

| **Components of exercise heart rate response/recovery** | **Hazard ratio (95% confidence interval)** | |
| --- | --- | --- |
|  | **Model 1** | **Model 2** |
| Resting heart rate (bpm) ^b^ | **1.07 (0.99, 1.17)** | **1.11 (1.02, 1.21) ^*^** |
| Peak heart rate (bpm) ^b^ | 1.05 (0.95, 1.15) | 1.09 (0.99, 1.19) |
| Recovery heart rate (bpm) ^b^ | **1.11 (1.01, 1.22) ^*^** | **1.15 (1.05, 1.27) ^**^** |
| Response ratio ^b^ | 0.94 (0.85, 1.03) | 0.93 (0.85, 1.02) |
| Recovery ratio ^b^ | 0.91 (0.81,1.01) | 0.90 (0.80, 1.01) |
| Heart rate response/recovery index ^b^ | 0.91 (0.82, 1.01) | 0.90 (0.81, 1.00) |

Model 1 was adjusted for sociodemographic variables (age, sex, education, ethnicity, and deprivation). Model 2 was further adjusted for lifestyle factors (physical activity, body mass index, smoking, and alcohol intake), cardiovascular disease risk score (high blood pressure, cholesterol, diabetes, ischemic heart disease, and peripheral vascular disease), and HR-control medications.

^a^ The competing event was defined as death occurring before follow-up assessment without a dementia diagnosis.

^b^ Per 1-SD increase.

^*^*P* <0.05, ^**^*P* <0.01.

**Table S2.** Characteristics of study participants by inclusion and exclusion status in the analytical sample.

|  | Total sample | Analytical sample | | |
| --- | --- | --- | --- | --- |
| Characteristics | (n=61,484) | Included (n=46,348) | Excluded (n=15,136) | P-value |
| Age, years | 56.36 (8.14) | 56.38 (8.12) | 56.32 (8.22) | 0.413 |
| Male, n (%) | 29,650 (48.21) | 22,912 (49.43) | 6738 (44.47) | <0.001 |
| College attendance, n (%) | 22,736 (36.97) | 18,201 (39.27) | 4,535 (29.93) | <0.001 |
| Resting HR, bpm | 71.15 (11.54) | 70.92 (11.46) | 71.86 (11.74) | <0.001 |
| Peak exercise HR, bpm | 113.50 (13.87) | 112.98 (13.68) | 115.10 (14.29) | <0.001 |
| Recovery HR, bpmd | 82.30 (13.84) | 81.85 (13.76) | 83.66 (13.98) | <0.001 |
| Response ratio | 1.62 (0.24) | 1.62 (0.23) | 1.63 (0.24) | <0.001 |
| Recovery ratio | 1.40 (0.18) | 1.40 (0.18) | 1.40 (0.19) | 0.023 |
| HRR index | 2.29 (0.58) | 2.29 (0.57) | 2.30 (0.61) | 0.113 |
| Incident dementia, n (%) | 718 (1.17) | 519 (1.12) | 199 (1.31) | 0.060 |

Data are mean (standard deviation), unless otherwise specified.

Abbreviations: HR, heart rate; HRR, heart rate response/recovery.


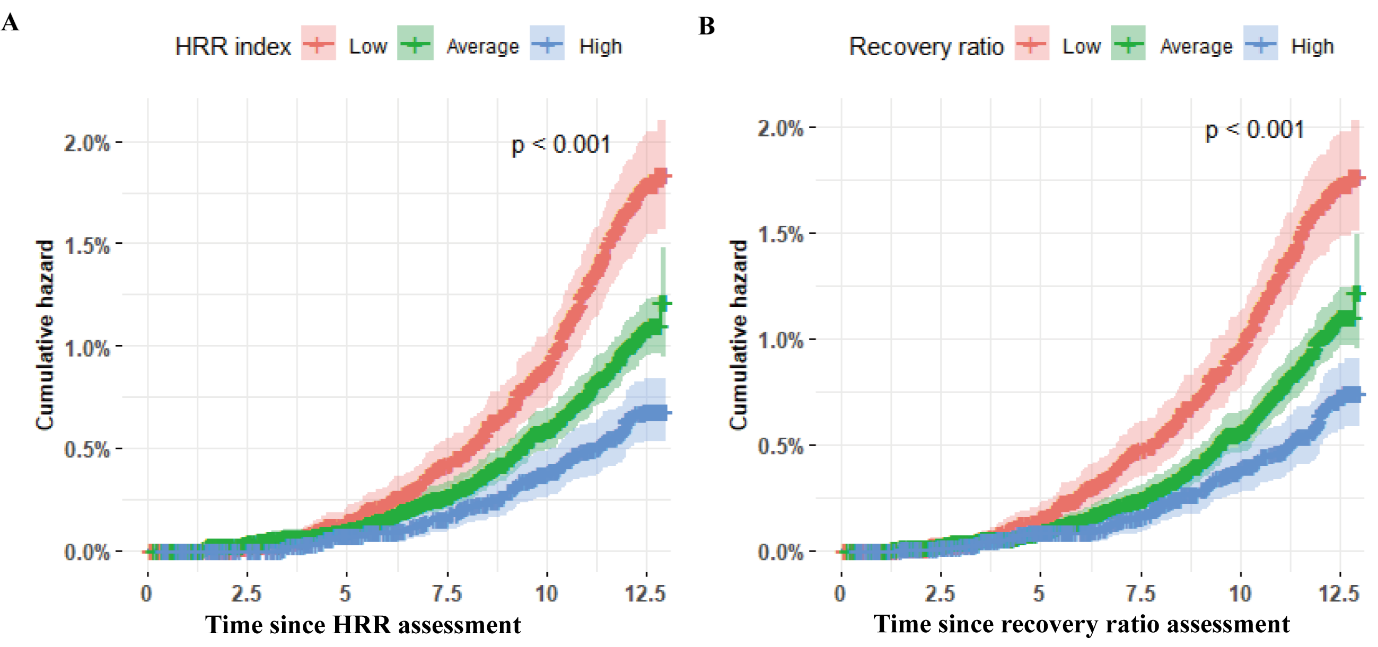


**Figure S1**. Cumulative risk for incident all-cause dementia over time by HRR index (A) and recovery ratio (B). Cumulative incidence plot (95% CI shaded) showing percentage of cohort with a first diagnosis of dementia over time in each of the HRR index groups and recovery ratio groups. HRR, heart rate response/recovery.

**Table S3.** Associations of individual components of exercise heart rate response/recovery index with incident dementia

| **Exercise HRR group** | **Hazard ratio (95% confidence interval)** | | | | | | | |
| --- | --- | --- | --- | --- | --- | --- | --- | --- |
|  | **All-cause dementia (n=519)** | |  | **Alzheimer’s Disease (n=232)** | |  | **Vascular dementia (n=85)** | |
|  | **Model 1** | **Model 2** |  | **Model 1** | **Model 2** |  | **Model 1** | **Model 2** |
| Resting HR |  |  |  |  |  |  |  |  |
| Continuous ^a^ | 1.08 (1.00, 1.18) | **1.12 (1.03, 1.22) ^**^** |  | 1.03 (0.90, 1.17) | 1.07 (0.94, 1.22) |  | 0.93 (0.75, 1.16) | 0.97 (0.78, 1.21) |
| Categorical ^b^ |  |  |  |  |  |  |  |  |
| Low  (40-63) | 1.00 (reference) | 1.00 (reference) |  | 1.00 (reference) | 1.00 (reference) |  | 1.00 (reference) | 1.00 (reference) |
| Average  (63-78) | 1.14 (0.92, 1.40) | 1.22 (0.99, 1.51) |  | 0.96 (0.71, 1.31) | 1.02 (0.75, 1.40) |  | 1.15 (0.70, 1.90) | 1.28 (0.77, 2.14) |
| High  (78-187) | 1.14 (0.89, 1.47) | 1.25 (0.96, 1.61) |  | 0.91 (0.63, 1.31) | 0.99 (0.68, 1.45) |  | 0.78 (0.41, 1.50) | 0.86 (0.44, 1.68) |
| *P* for trend | 0.27 | 0.08 |  | 0.62 | 0.75 |  | 0.52 | 0.97 |
| Peak HR |  |  |  |  |  |  |  |  |
| Continuous ^a^ | 1.05 (0.96, 1.15) | **1.10 (1.00, 1.20) ^*^** |  | 0.95 (0.83, 1.09) | 0.98 (0.86, 1.12) |  | 0.90 (0.72, 1.12) | 0.98 (0.79, 1.22) |
| Categorical ^b^ |  |  |  |  |  |  |  |  |
| Low  (61-104) | 1.00 (reference) | 1.00 (reference) |  | 1.00 (reference) | 1.00 (reference) |  | 1.00 (reference) | 1.00 (reference) |
| Average  (105-122) | 1.02 (0.84, 1.24) | 1.11 (0.91, 1.35) |  | 0.81 (0.61, 1.08) | 0.86 (0.64, 1.15) |  | 0.66 (0.41, 1.05) | 0.75 (0.46, 1.22) |
| High  (123-200) | 1.15 (0.89, 1.24) | 1.23 (0.95, 1.60) |  | 0.98 (0.67, 1.43) | 1.02 (0.70, 1.50) |  | 0.84 (0.45, 1.58) | 0.94 (0.50, 1.78) |
| *P* for trend | 0.33 | 0.11 |  | 0.60 | 0.60 |  | 0.31 | 0.85 |
| Recovery HR |  |  |  |  |  |  |  |  |
| Continuous ^a^ | **1.12 (1.03, 1.22) ^*^** | **1.17 (1.06, 1.28) ^***^** |  | 0.98 (0.86, 1.12) | 1.02 (0.89, 1.17) |  | 0.92 (0.74, 1.14) | 1.03 (0.76, 1.20) |
| Categorical ^b^ |  |  |  |  |  |  |  |  |
| Low  (40-72) | 1.00 (reference) | 1.00 (reference) |  | 1.00 (reference) | 1.00 (reference) |  | 1.00 (reference) | 1.00 (reference) |
| Average  (73-91) | 1.06 (0.85, 1.31) | 1.15 (0.93, 1.44) |  | 0.92 (0.68, 1.25) | 0.99 (0.73, 1.35) |  | 0.82 (0.50, 1.35) | 0.93 (0.56, 1.56) |
| High  (92-146) | **1.34 (1.05, 1.70) ^*^** | **1.47 (1.14, 1.89) ^**^** |  | 0.88 (0.61, 1.28) | 0.96 (0.65, 1.41) |  | 0.92 (0.51, 1.65) | 1.02 (0.55,1.88) |
| *P* for trend | 0.02 | p <0.01 |  | 0.50 | 0.84 |  | 0.74 | 0.97 |
| Response ratio |  |  |  |  |  |  |  |  |
| Continuous ^a^ | 0.93 (0.85, 1.02) | 0.93 (0.84, 1.02) |  | 0.92 (0.79, 1.06) | 0.90 (0.78, 1.04) |  | 0.94 (0.74, 1.18) | 0.97 (0.77, 1.22) |
| Categorical ^b^ |  |  |  |  |  |  |  |  |
| Low  (1-1.45) | 1.00 (reference) | 1.00 (reference) |  | 1.00 (reference) | 1.00 (reference) |  | 1.00 (reference) | 1.00 (reference) |
| Average  (1.45-1.75) | 0.92 (0.76, 1.11) | 0.93 (0.77, 1.14) |  | 0.94 (0.70, 1.25) | 0.93 (0.70, 1.25) |  | 0.74 (0.46, 1.18) | 0.80 (0.49, 1.29) |
| High  (1.75-3.87) | 0.81 (0.62, 1.05) | 0.80 (0.61, 1.05) |  | 0.84 (0.57, 1.25) | 0.81 (0.54, 1.22) |  | 0.87 (0.47, 1.61) | 0.93 (0.49, 1.76) |
| *P* for trend | 0.11 | 0.13 |  | 0.40 | 0.66 |  | 0.47 | 0.33 |

Model 1 was adjusted for sociodemographic variables (age, sex, education, ethnicity, and deprivation). Model 2 was additionally adjusted for lifestyle factors (physical activity, body mass index, smoking, and alcohol intake), cardiovascular disease risk score (high blood pressure, cholesterol, diabetes, ischemic heart disease, and peripheral vascular disease), and use of heart rate control medications.

^a^ Per 1-SD increase.

^b^ HRR index was categorized into low (≤25 percentile), average (25-75 percentile), and high (>75 percentile) groups.

^*^*P* < 0.05, ^**^*P* < 0.01, ^***^*P* < 0.001.

**Table S4.** Subgroup analyses of dementia risk with recovery ratio and the heart rate response/recovery index.

|  | No. of cases | No. of participants | Hazard ratio (95% confidence interval) | | | | |
| --- | --- | --- | --- | --- | --- | --- | --- |
|  |  |  | HRR index | *p* ^interaction^ |  | Recovery ratio | *p* ^interaction^ |
| All participants | 519 | 46,348 | **0.90 (0.81, 1.00) ^*^** |  |  | **0.89 (0.80, 0.99) ^*^** |  |
| Sex |  |  |  |  |  |  |  |
| Male | 316 | 22,912 | 0.68 (0.59, 0.79) ^***^ | 0.07 |  | 0.67 (0.58, 0.78) ^***^ | 0.03 |
| Female | 203 | 23,436 | 0.84 (0.71, 0.99) ^*^ |  |  | 0.87 (0.74, 1.02) |  |
| Age |  |  |  |  |  |  |  |
| <65 years | 218 | 37,799 | 0.81 (0.68, 0.95) ^*^ | 0.20 |  | 0.79 (0.68, 0.93) ^**^ | 0.29 |
| ≥65 years | 301 | 85,49 | 0.87 (0.75, 1.00) |  |  | 0.88 (0.76, 1.01) |  |
| Education |  |  |  |  |  |  |  |
| Either college-level | 371 | 28,147 | 0.73 (0.64, 0.83) ^***^ | 0.73 |  | 0.75 (0.66, 0.85) ^***^ | 0.97 |
| non-college-level | 148 | 18,201 | 0.79 (0.65, 0.96) ^*^ |  |  | 0.77 (0.63, 0.94) ^**^ |  |
| Physical activity level |  |  |  |  |  |  |  |
| Lower | 307 | 29,741 | 0.73 (0.64, 0.85) ^***^ | 0.74 |  | 0.76 (0.66, 0.88) ^***^ | 0.39 |
| Higher | 212 | 16,607 | 0.76 (0.65, 0.90) ^**^ |  |  | 0.75 (0.63, 0.88) ^***^ |  |
| CVD risks |  |  |  |  |  |  |  |
| None | 233 | 30,486 | 0.68 (0.58, 0.80) ^***^ | 0.70 |  | 0.67 (0.57, 0.79) ^***^ | 0.10 |
| Any | 286 | 15,862 | 0.77 (0.66, 0.89) ^***^ |  |  | 0.79 (0.68, 0.92) ^**^ |  |

“Lower/Higher” was dichotomized based on the median values for physical activity.

^a^ Per 1-SD increase.

Abbreviation: CVD, cardiovascular disease.

^*^P < 0.05, ^**^P < 0.01, ^***^P < 0.001.
